# Supplementary material for: Causal interplay between lactose intolerance and gut microbiota: a combined bidirectional Mendelian randomization and in vivo validation study
Source: Front Nutr. 2026 Jun 1;13:1803337. doi: 10.3389/fnut.2026.1803337 (PMC13265576; doi:10.3389/fnut.2026.1803337)
Supplement: Supplementary file 1 [file Data_Sheet_1.zip › supplementary materials/Reverse/Batch analysis results 20250616220940/funnel chart/finngen_R12_E4_LACTONAS.ebi-a-GCST90027490.pdf]

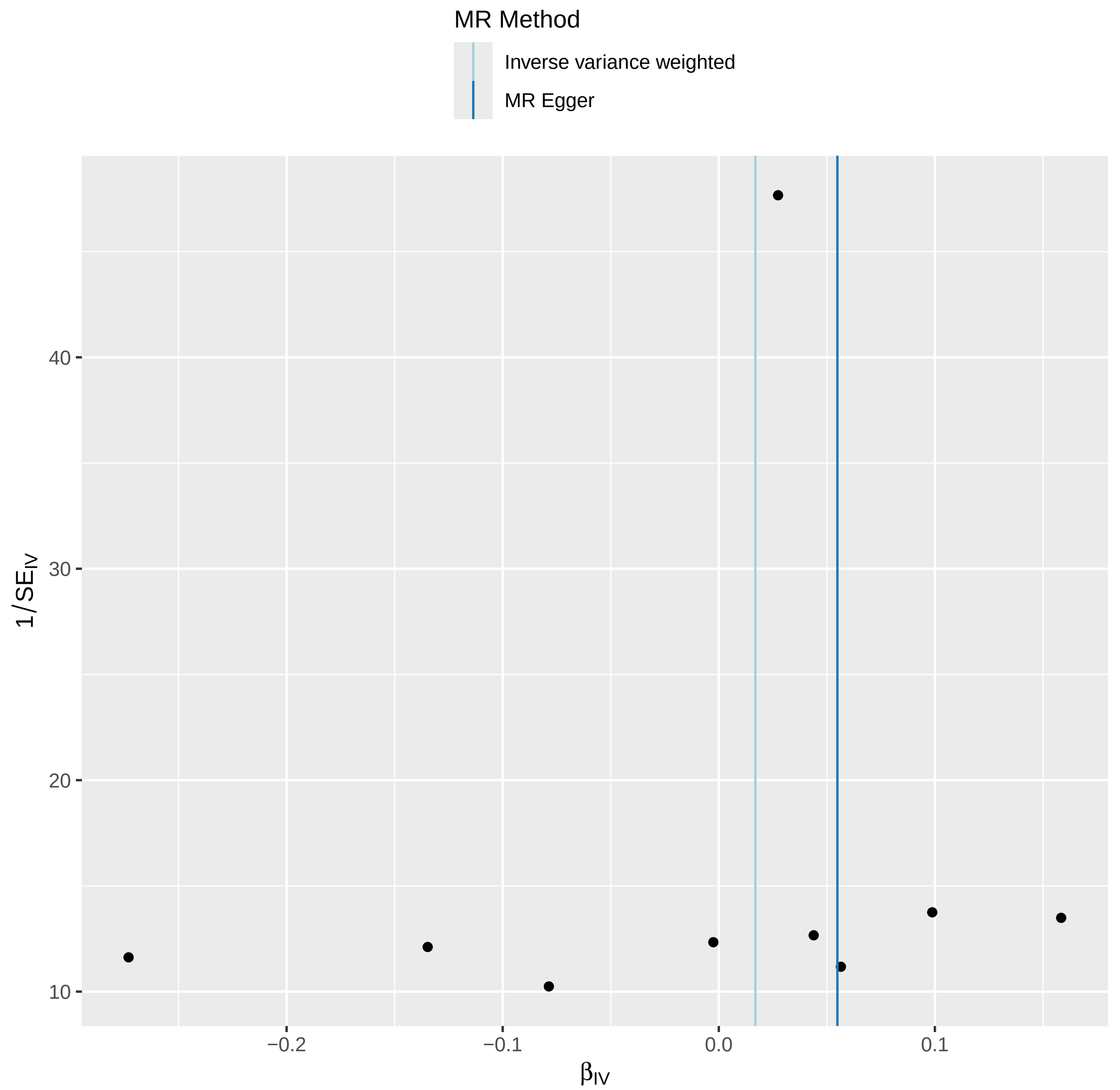

MR Method
Inverse variance weighted
MR Egger
40
30
20
10
1/SE_IV
−0.2
−0.1
0.0
0.1
β_IV
